# Framing the Issue

**DOI:** 10.1093/geroni/igaa057.2547

**Published:** 2020-12-16

**Authors:** Brian Lindberg

**Affiliations:** GSA, Washington, District of Columbia, United States

## Abstract

GSA's Public Policy Advisor will lead a discussion of legislative activities related to elder abuse, neglect, and exploitation during the past year. The panel will examine successes and failures and what may still be possible in the lame duck session and the next Congress.

